# Putting High-Index Cu on the Map for High-Yield, Dry-Transferred CVD Graphene

**DOI:** 10.1021/acsnano.2c09253

**Published:** 2023-01-03

**Authors:** Oliver J. Burton, Zachary Winter, Kenji Watanabe, Takashi Taniguchi, Bernd Beschoten, Christoph Stampfer, Stephan Hofmann

**Affiliations:** † Department of Engineering, University of Cambridge, CambridgeCB3 0FA, United Kingdom; ‡ 2nd Institute of Physics A and JARA-FIT, RWTH Aachen University, 52074Aachen, Germany; § Research Center for Functional Materials, National Institute for Materials Science, 1-1 Namiki, Tsukuba, Ibaraki305-0044, Japan; ∥ International Center for Materials Nanoarchitectonics, National Institute for Materials Science, 1-1 Namiki, Tsukuba, Ibaraki305-0044, Japan; ⊥ Peter Grünberg Institute (PGI-9), Forschungszentrum Jülich, 52425Jülich, Germany

**Keywords:** CVD, graphene, single crystal, dry transfer, data science, 2D material, high electron mobility

## Abstract

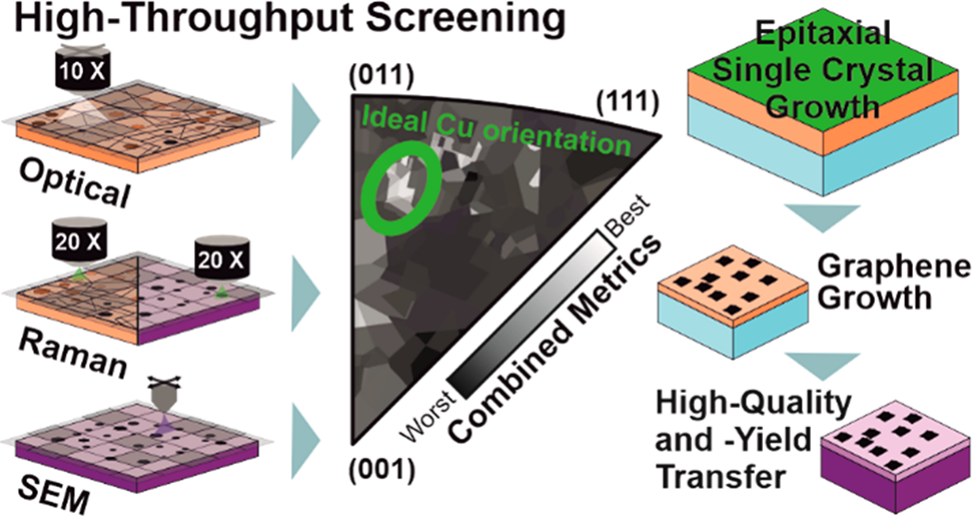

Reliable, clean transfer and interfacing of 2D material layers are technologically as important as their growth. Bringing both together remains a challenge due to the vast, interconnected parameter space. We introduce a fast-screening descriptor approach to demonstrate holistic data-driven optimization across the entirety of process steps for the graphene–Cu model system. We map the crystallographic dependences of graphene chemical vapor deposition, interfacial Cu oxidation to decouple graphene, and its dry delamination across inverse pole figures. Their overlay enables us to identify hitherto unexplored (168) higher index Cu orientations as overall optimal orientations. We show the effective preparation of such Cu orientations via epitaxial close-space sublimation and achieve mechanical transfer with a very high yield (>95%) and quality of graphene domains, with room-temperature electron mobilities in the range of 40000 cm^2^/(V s). Our approach is readily adaptable to other descriptors and 2D material systems, and we discuss the opportunities of such a holistic optimization.

2D materials (2DMs), spearheaded by graphene, continue to be an extremely powerful platform for the scientific discovery of ever more complex properties and functionalities. There is, however, a widening gap between individual demonstrator or “hero” devices and what is possible to reproducibly fabricate with scalable methodologies. This presents a key bottleneck for translation to technology, in particular for higher-value-added applications such as integrated sensors, flexible high-frequency electronics, or broad-band optoelectronics, as highlighted across current technology roadmaps.^1−3^ Chemical vapor deposition (CVD) has matured as the leading technique to scalable crystal growth of monolayer/few-layer graphene,^4−6^ and the as-grown synthetic material has reached the quality (as defined by electron mobility measurements) set by exfoliation from bulk crystals.^7−9^ Most applications involve transfer away from the growth substrate, and such transfer and handling technology is thus an integral part of the scalable CVD approach.^3,10^ Given the notoriously vast, combined parameter space, to date graphene CVD and transfer optimization have largely been explored in separation, with all early focus being on the initial synthesis parameters and utilization of catalytic enhancement via transition metals such as Cu.^11,12^ Such catalytic growth of graphene has a high dependence on Cu facet orientation, whereby most recent growth studies converged on using Cu(111),^13−18^ due to the ease of production of such a low-index orientation both via foil crystallization and epitaxial metalization approaches, as well as enabling a uniform epitaxial alignment of graphene. In order to promote transferability, the graphene–Cu interaction must be weakened postgrowth, to decrease graphene adhesion enough for clean, reliable delamination and for the Cu template to be reused. An efficient approach for this is interfacial Cu oxidation.^8,19,20^ Such a postgrowth process is also known to have a high dependence on the Cu surface orientation.^21^ A common observation across many different oxidation approaches is the low achievable rate of oxidation of Cu(111) underneath graphene.^21−24^ This indicates the shortcomings of the current sequential optimization approach, where graphene on the growth substrate might be “high quality” but subsequent transfer is compromised, and so will the device yield and achievable properties.

Here, we use a fast-screening descriptor approach to demonstrate a holistic, combined optimization approach across the entirety of process steps for growth and transfer for the graphene–Cu model system. We focus on enabling an efficient dry transfer of CVD graphene islands, as this is currently a much sought after capability and a critical stage to address the demand for reproducible, high-yield device fabrication relying on cleanly interfaced 2D material stacks. We systematically track and study 1000s of graphene islands on over 100 crystallographic Cu orientations and plot quality descriptors for each process step across inverse pole figures (IPFs). This representation allows us to overlay IPFs to identify higher-index Cu orientations that are best suited for the combined overall process. We employ an epitaxial close-space sublimation approach^15^ to exclusively create optimum Cu(168) orientations, establishing translation to a scalable pathway for graphene island growth and transfer at high (>95%) yield. After h-BN encapsulation, we demonstrate room-temperature electron mobilities of over 40 × 10^3^ cm^2^/(V s) at 1 × 10^12^ cm^–2^ and average Raman 2D line widths of ∼16 cm^–1^. We find this approach is extremely powerful to navigate and gain key insights across these notoriously large, interconnected parameter spaces and is readily adaptable to many other catalyst–2D material systems while being expandable to include future relevant descriptors.

## Results and Discussion

**Figure 1:**
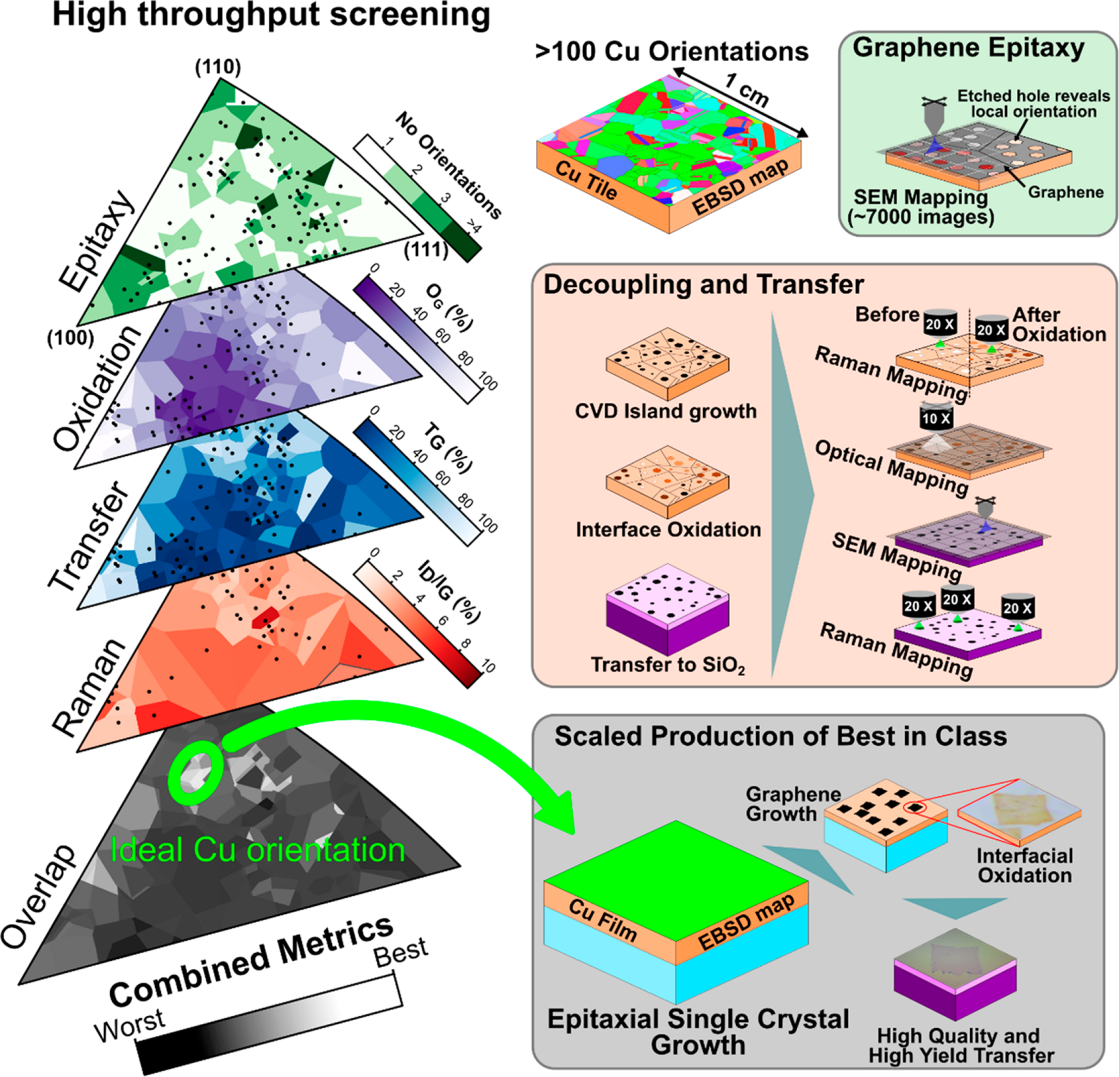
Schematic summary of the holistic, high-throughput characterization methodology and epitaxial production of select orientations. Each key screening parameter is mapped on an inverse pole figure representing the catalyst crystallographic orientation, with the combined metrics (an overlay of these screening parameters) revealing the optimum Cu orientation. These metrics include the graphene alignment, interfacial oxidation propensity, transferred percentage, and Raman characterization for individual graphene islands. The optimum Cu orientation is then reproducibly created using an epitaxial growth platform, enabling high-yield graphene island transfer.

We utilize two different catalyst preparation methods, as outlined in Figure 1. First, we use polycrystalline, 1 × 1 cm^2^ Cu tiles (see Experimental Details) exhibiting a large number (>100) of different Cu crystallographic orientations, each of them sufficiently large (>100 μm), allowing for the effective high-throughput characterization of graphene growth, interfacial Cu oxidation to decouple graphene, and its mechanical delamination. We fully map the surface crystal orientations of the Cu tiles by electron backscatter diffraction (EBSD). For each process step we identify a key quality metric (Figure 1; each of which is discussed in detail below) that can be effectively, automatically mapped and compiled as an IPF. By overlaying individual process step IPFs, the use of polycrystalline Cu tiles thus allows the identification of Cu orientations that are overall most promising throughout the growth and transfer parameter space. To selectively work with as-identified optimum Cu orientations, we employ an epitaxial close-space sublimation approach^15^ as a second catalyst preparation method that enables the scalable production of single-crystal metal templates. We use graphene islands grown on these single-crystal templates to characterize the graphene in terms of the reproducible transfer yield of multiple islands and through the fabrication of encapsulated test devices to confirm a high-quality material.

**Figure 2:**
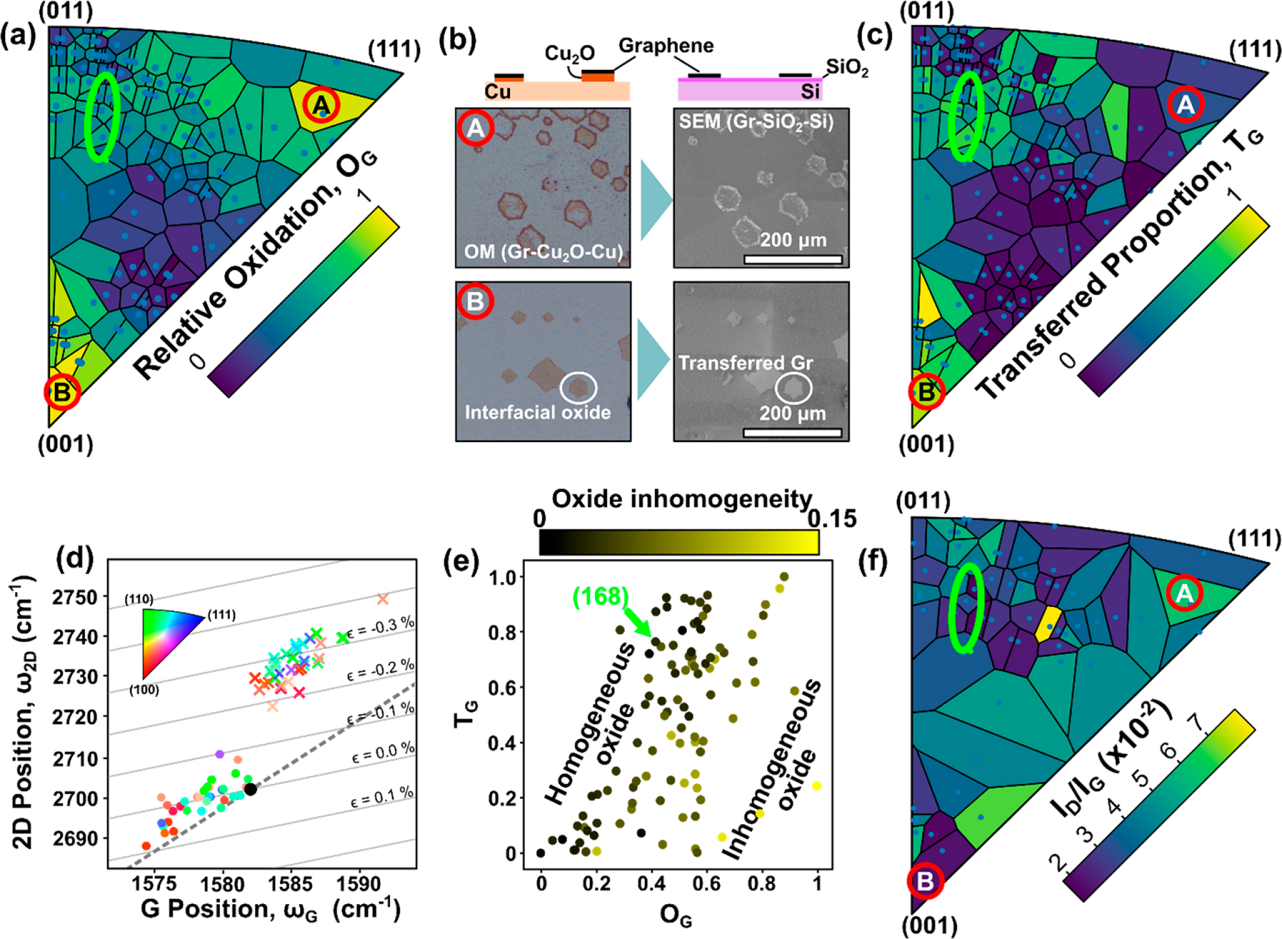
Mapping of interfacial oxidation, graphene transfer yield, and quality after transfer onto SiO_2_-Si as defined by Raman *I*_D_/*I*_G_ peak ratios. (a) An IPF showing the relative oxidation of the Cu–Gr interface, *O*_G_, dependent on the crystallographic Cu orientation. (b) Schematic illustration of the ordering of the Gr–Cu_2_O–Cu and Gr–SiO_2_–Si samples with example images from OM and SEM shown below, labeled A and B with corresponding identifiers on the IPFs. (c) An IPF showing the fraction of transferred graphene, *T*_G_, from Cu after oxidation as a function of Cu orientation. (d) The mean Raman 2D peak position against the G peak position for graphene on Cu before (× in different colors) and after (● in different colors) oxidation with lines of constant strain labeled and a line of constant doping shown for reference and the pristine point (the point with no strain or doping) shown as a large black point. The color of this scatter plot is linked to the crystal orientation of the Cu where the graphene measurement was taken, according to the IPF inset. (e) Scatter plot of *T*_G_ vs the relative oxidation, *O*_G_, with the color of each data point mapping to the inhomogeneity of the relative oxidation (see Experimental Details). (f) An IPF of the *I*_D_/*I*_G_ Raman peak ratio of Gr after mechanical delamination and transfer onto a SiO_2_ substrate as a function of Cu orientation. The blue dots in the IPFs in this figure represent the average crystallographic orientation of the Cu facet as measured by EBSD, and the green ellipse represents the ideal region around Cu(168).

In this work, we focus on the mechanical delamination of graphene from its growth substrate. To generate a significant number of data points for analysis, we focus on individually grown graphene islands on a polycrystalline Cu catalyst. Motivated by prior work, we use saturated water vapor exposure to promote interfacial Cu oxidation^8,21,25^ prior to delamination with a PVA film (see Experimental Details). Figure 2 connects the relative oxidation level beneath graphene islands to the yield of their mechanical delamination and quality of as-transferred graphene on the SiO_2_ support. The clear optical contrast due to Cu oxidation allows us to employ optical microscopy (OM) and introduce a quantitative parameter *O*_G_. Here, *O*_G_ represents the normalized mean relative Cu oxidation contrast of the areas beneath all graphene islands on a given Cu orientation (see Interfacial Oxidation in Experimental Details). Figure 2a shows an IPF for *O*_G_ and demonstrates the variation in interfacial oxidation as a function of Cu orientation. The interfacial oxidation was further characterized by X-ray photoelectron spectroscopy (XPS) and imaging ellipsometry (IE) to confirm that the high-throughput screening via *O*_G_ is indeed a meaningful metric (See Figures S5 and S7 in the Supporting Information). To capture transfer yield, we use scanning electron microscopy (SEM) mapping and introduce a quantitative parameter *T*_G_ (See Mechanical Delamination in Experimental Details) that reflects the average proportion of graphene islands that are transferred onto SiO_2_ via the mechanical delamination process. Figure 2c presents *T*_G_ from the Cu growth substrate to a Si/SiO_2_ substrate as an IPF.

Figure 2e presents a scatter plot of *T*_G_ vs *O*_G_ to highlight the relationship between these two parameters. The data points are colored to highlight oxide inhomogeneity (see Interfacial Oxidation in Experimental Details), a measure of the variation of the interfacial oxide from the mean with lower values meaning that the oxide is more homogeneous (i.e., exhibiting a more uniform contrast). Our data identify a clear underlying trend: the more interfacial Cu oxide and the more homogeneous that oxide, the higher the proportion of successfully transferred graphene. It is notable that our measured relative oxide thicknesses and *O*_G_ trends across the IPF are consistent with previous literature,^21^ despite different oxidation and exposure conditions. This indicates that the trends we show are representative across a reasonably large set of potential oxidation conditions. Example OM and SEM images are labeled A and B (approximately Cu(111) and Cu(100), respectively) in Figure 2b, with corresponding locations marked on Figure 2a,c,f, highlighting how more oxidized Cu regions link to a higher success rate for graphene transfer. It is noted in example data A that while a visible degree of oxidation can be seen beneath the center of some graphene domains, these areas are not successfully transferred, while the more oxidized regions at the edge of the graphene islands are. This implies one, or a combination, of three scenarios: (1) that there is a threshold oxide thickness requirement for decoupling and delamination, (2) that the oxide inhomogeneity has a higher spatial frequency than the resolution of the imaging techniques used, or (3) that the oxide in the center of these graphene domains is different from that of the outside, i.e. Cu_2_O vs CuO, and couples strongly to the graphene. We can rule out the last scenario, as XPS on Cu(111), similar to the orientation in question, and on all other Cu facets measured (see Figure S7 in the Supporting Information) shows a lack of Cu^2+^ at the surface. This implies that the dominant oxide formed at the Cu–graphene interface is Cu_2_O. While example areas A and B demonstrate the importance of the oxide homogeneity and presence, they also highlight the complexity of the system. There are different oxidation mechanisms that have significant variations in both rate and propensity of lateral oxide propagation. Example A in Figure 2b demonstrates this lack of propensity with only a thick oxide observed at the edge of the graphene islands. Single-crystal-prepared Cu(111) shows this lack of oxidation as well, with our tests (similar graphene islands on single-crystal Cu(111) in the same humidified environment described in Experimental Details) showing no propagation of oxidation beneath graphene grown on Cu(111) even after several weeks of oxidation. This holds true for many Cu orientations.^26^ This lack of oxidation is consistent with prior literature, which suggests that Cu(111) inhibits extended oxidation beneath graphene^22^ and speculates that this links to the close commensurate matching and thus coupling of in-plane graphene and Cu(111).^27^ However, by examining the Cu regions not covered by graphene (see Figure S13 in the Supporting Information), we reveal that there is in general a strong correlation (0.770 Pearson correlation coefficient) between the oxidation of Cu facets beneath the graphene and of the uncovered Cu. This strong correlation implies that most of the variation in oxidation between facets under graphene is also seen on bare Cu; thus, decreases in propensity to oxidation are unlikely to be the result of graphene.

Figure 2f shows the graphene D to G Raman peak intensity ratio (*I*_D_/*I*_G_) as a function of Cu orientation as an IPF plot. The *I*_D_/*I*_G_ ratio is a commonly used metric in the literature, with higher values corresponding to a higher defect density in graphene.^28^Figure 2f shows tendencies similar to those of Figure 2c: areas with lower *T*_G_ have a higher *I*_D_/*I*_G_, or statistically speaking *I*_D_/*I*_G_ vs *T*_G_ has a Pearson correlation coefficient of −0.38, implying that Cu orientations with a high *T*_G_ tend to yield a graphene film with lower defect densities after transfer. We postulate that the variation of *I*_D_/*I*_G_ with Cu orientation corresponds to defects in the film as a result of cracks and holes formed through the graphene transfer process, rather than any intrinsic variation in the quality of the graphene as grown on different Cu orientations. This cracking and its effects on Raman spectroscopy measurements can be seen in Figure S3d in the Supporting Information. Figure 2d plots the 2D Raman peak position ω_2D_ against ω_G_ for a range of Cu orientations, showing a strong difference between the Raman peak positions, which have been correlated to strain,^29^ before and after oxidation. These peak shifts imply that as-grown graphene on all measured bare Cu facets is under compressive strain, which upon interfacial Cu oxidation reduces or shifts to tensile strain.^20^ This shift is consistent with the volume expansion upon Cu oxidation,^30^ given a Pilling–Bedworth ratio of 1.7 for Cu_2_O. It is noted that we have adjusted the pristine point (i.e., the value of ω_2D_ and ω_G_ representing no strain or doping) for the laser wavelength used (457 nm) according to the Raman peak dispersion experimentally determined in the literature.^31,32^

An analysis of the 2D and G Raman peak widths (Γ) shows that initially there is a wide range of Γ_2D_ and Γ_G_ before oxidation, narrowing after oxidation to a much smaller region of higher average Γ_2D_ and Γ_G_ (see Figure S3a in the Supporting Information). The literature has previously established different Γ_2D_ values for graphene on different crystallographic orientations of Cu, which is notably reflected in Figure S3a in the Supporting Information with Cu(111) having a broader 2D band than both Cu(110) and Cu(100).^33^ Previous experiments have linked the increase in Γ_2D_ to an increase in the magnitude of nanoscale strain variations.^34^ This correlates well with our measurements of the surface microstructure of as-oxidized Cu facets beneath graphene layers, which are microscopically rougher than the initial metallic Cu at the interface (see an example in Figure S9 in the Supporting Information), consistent with reports across the literature.^35,36^ The shift to lower Raman peak positions and increase in widths imply that the graphene is moving from a region of consistent compressive strain to a region of tensile strain with larger variations in local strain. We interpret this as the graphene being detached from its relatively strong coupling to the bare Cu to rest on a rougher Cu oxide surface, which then facilitates mechanical delamination. The Γ_2D_ values of the graphene on Cu here show significantly higher values than those after transfer onto the SiO_2_–Si substrate (see Figure 2c). However, we observe no clear dependence on Cu surface orientation between the Γ_2D_ values before and after mechanical delamination, implying that the Γ_2D_ value measured before transfer is a poor predictor or quality metric of any graphene characteristics after transfer onto another substrate. The data shown in Figure 2 shows that for the graphene–Cu system reproducible mechanical graphene delamination with a low defect density requires effective full and homogeneous oxidation of the buried Cu interface to graphene.

**Figure 3:**
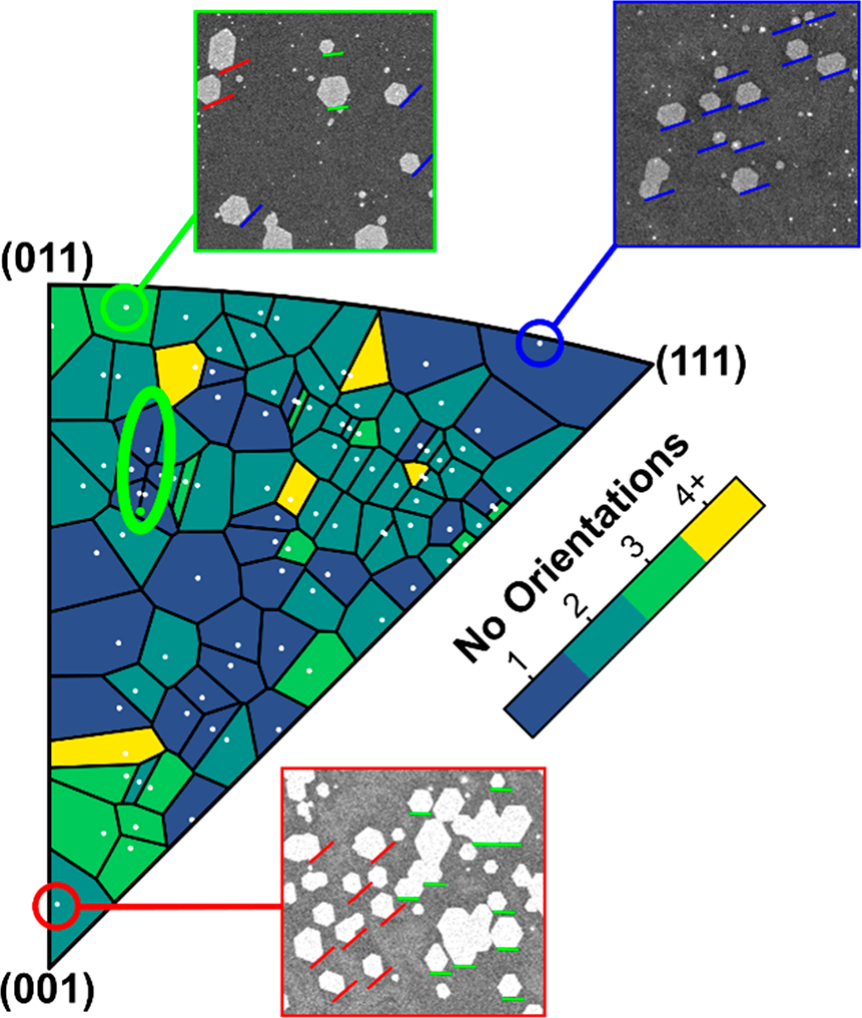
An IPF showing the number of graphene orientations grown on each measured Cu orientation (white points correspond to mean EBSD measurements for that facet). The SEM images show etched holes in graphene on select Cu orientations indicated on the IPF; the number of orientations of graphene etch holes found on each Cu facet is indicated by the color bar. The green ellipse highlights the region around Cu(168).

Given the strong intercalation and growth dependence on crystallographic alignment, the characterization of different potential graphene island orientations is important. We use a postgrowth Ar/H_2_ gas mixture after the graphene growth process to etch small hexagonal holes into a graphene film, locally exposing zigzag edges of graphene.^15^ Analyzing their orientation allows for an effective mapping of the local crystallographic orientation of the graphene. By combining 6515 SEM images, detecting and measuring the orientation of these zigzag-edged holes, and determining their location (383676 found holes) across the tiled Cu sample, we compiled Figure 3 an IPF map of the number of orientations of graphene found on each Cu facet orientation (see Figure S4 in the Supporting Information for additional details). Given our findings of Figure 2, it is highly preferred for the CVD process to lead to a single, uniform graphene island alignment. This has been shown to be also a prerequisite for single-crystalline graphene films,^37−40^ and most of the literature has thus focused on Cu(111). Our data for the low-index Cu orientations are consistent with prior literature: there are three orientations of graphene on ∼Cu(110),^41^ two orientations of graphene on Cu facets tending toward (100),^38,39^ and a single orientation of graphene grown on Cu(111).^38,40^Figure 3 shows, though, that there are a number of higher-index Cu orientations which also give a single graphene orientation. A direct overlay with IPFs in Figure 2 (shown in Figure 1) particularly motivates the cluster of higher index orientations around Cu(168). CVD graphene on these facets shows not only a single epitaxial orientation but also among the lowest *I*_D_/*I*_G_ ratios and Γ_2D_ widths (see Figure S6 in the Supporting Information for facet selection information), as well as high *O*_G_ and high *T*_G_.

**Figure 4:**
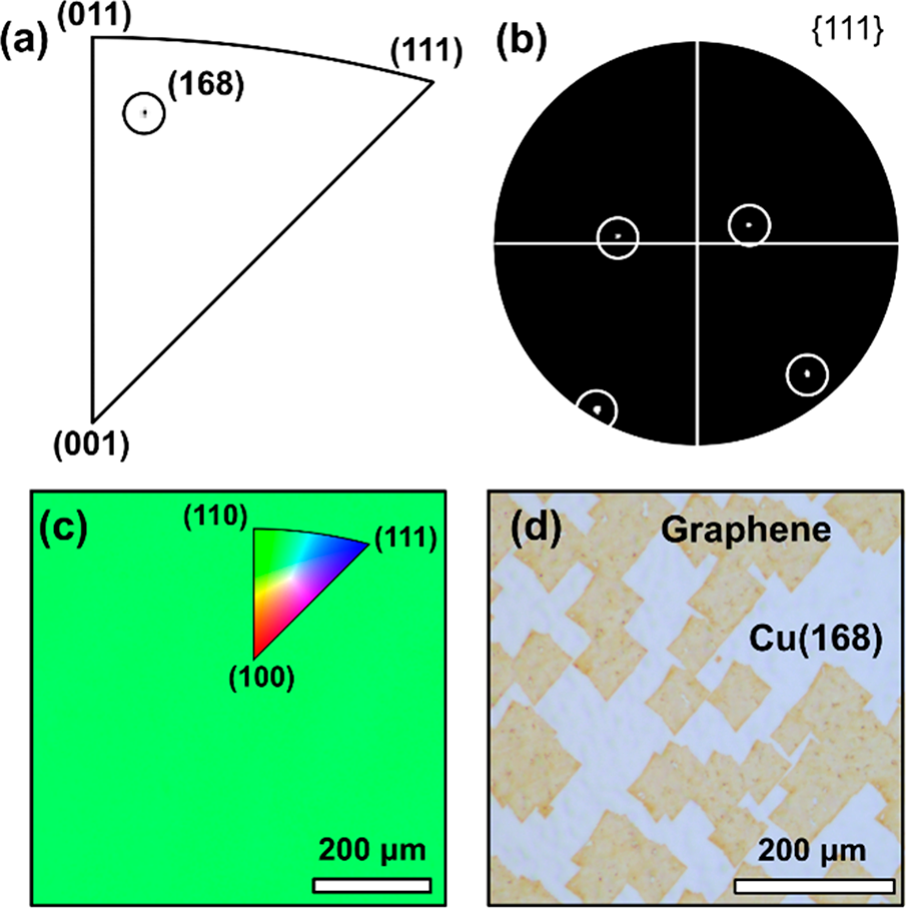
Characterization of epitaxial close-spaced sublimation Cu(168) film deposition on MgO(168) substrates. (a) A density IPF of the spatially mapped EBSD data. (b) A {111} pole figure of the EBSD data. (c) An IPF colored spatial map, with the inset showing a map of color to the crystallographic orientation. (d) An OM image showing the graphene islands (orange) after the interfacial oxidation.

Having identified orientations around Cu(168) as the optimum surface for growth and transfer using polycrystalline Cu tiles, Figure 4 shows that such Cu orientations can be selectively prepared via epitaxial Cu growth on MgO substrates. We employ an epitaxial close-spaced sublimation (CSS) approach that we previously introduced for single-crystal Cu(111) wafer growth (see Experimental Details).^15^ This allows scalable, cost-efficient epitaxial metalization at comparatively high rates and can be seamlessly combined with the graphene CVD process. Figure 4a–c shows the results of EBSD mapping and analysis of approximately 10 μm thick CSS Cu(168) films on centimeter-sized MgO(168). The IPF shows the creation of Cu(168), the {111} pole figure demonstrates that there is only one in-plane orientation of Cu(168), and the spatially resolved IPF map shows that this is consistent over large areas; these all confirm that the Cu films are single crystals over the analyzed ∼1 × 1 mm^2^ region. The absence of any thermal grooving observed by OM is further consistent with the single-crystal nature of as-grown epitaxial Cu. Figure 4d shows a representative OM image of graphene islands grown on such epitaxial Cu(168) after oxidation. Consistent with Figure 3, we observe a single graphene island orientation. Consistent with Figure 2, we observe homogeneous interfacial oxidation.

**Figure 5:**
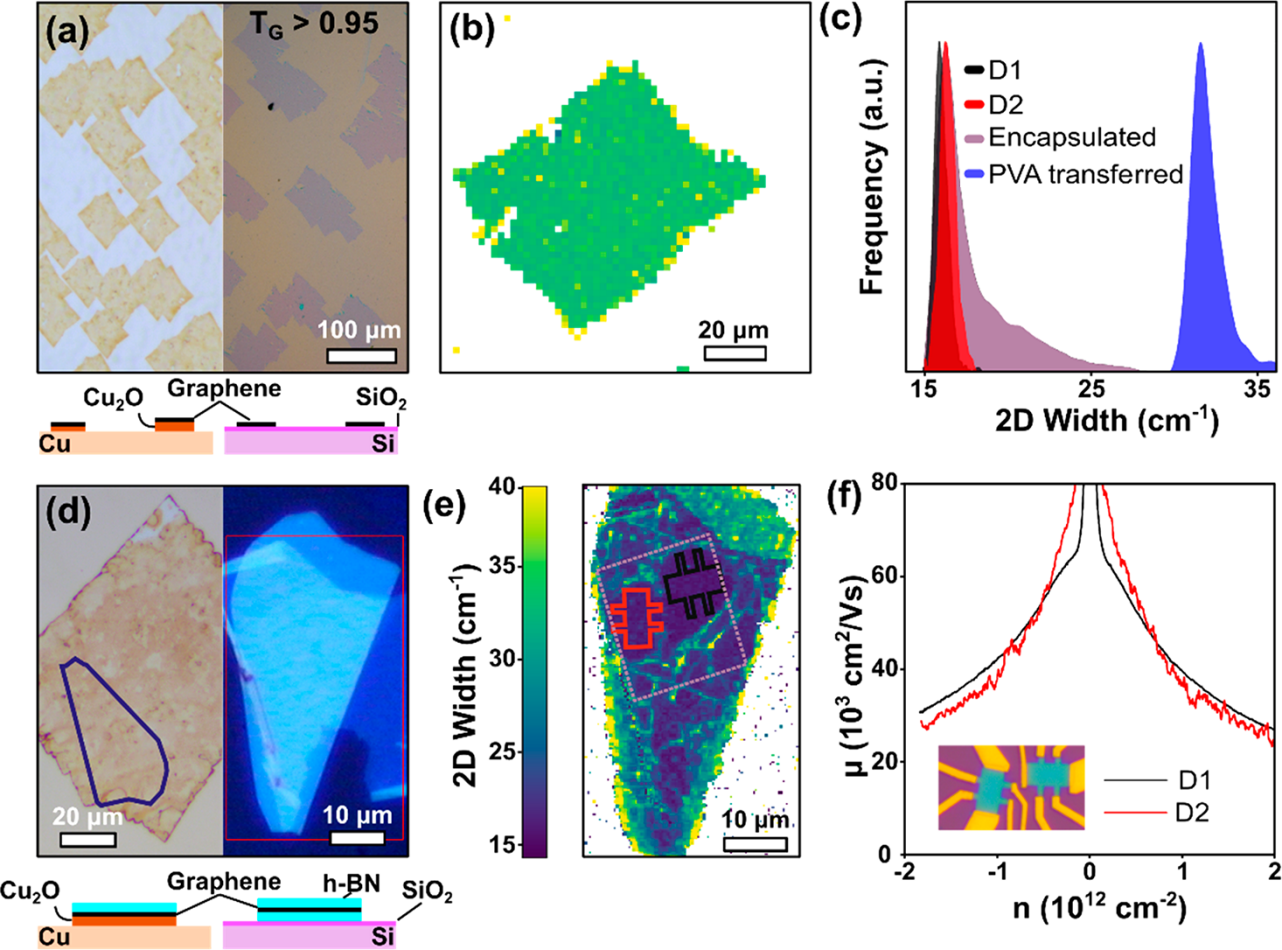
Yield and quality measurements of CVD graphene grown on CSS-deposited Cu(168). (a) OM images of the graphene islands before (left) and after (right) transfer with PVA onto Si/SiO_2_. The lower schematic shows the ordering of materials from a side view. (b) A spatially resolved map of the Raman 2D peak width of the graphene on Si/SiO_2_ shown in (a), with the color map being the same as that in (e). (c) A histogram of Raman 2D peak width of the data in (b) shown in blue and the data from (e) shown in black, red, and purple corresponding to the signal from the shapes outlined in (e). (d) OM images before and after encapsulation of the graphene in h-BN flakes, with schematic images showing ordering below. (e) A Raman 2D width map of the encapsulated region shown in (d), with regions used to make devices outlined. (f) The mobility as a function of carrier concentration at room temperature for both devices, with the line color corresponding to the respective regions in (e).

After finding the ideal growth–oxidation–transfer path through intersecting the respective parameter spaces, we use two simultaneous approaches to probe (1) the reproducible yield and (2) the quality of the graphene islands from Cu(168): (1) a PVA style transfer identical with that used above for the mechanical delamination from polycrystalline Cu tiles and (2) mechanical dry-delamination of graphene with exfoliated h-BN crystals to fabricate encapsulated Hall-bar devices. This combined approach allows us to quantify process reproducibility in terms of *T*_G_ and quality in terms of Raman spectroscopy and Hall mobilities. Figure 5a shows the graphene after oxidation on epitaxial CSS Cu(168) and after transfer on Si/SiO_2_. The optical contrast for the former highlights full and homogeneous Cu oxidation underneath the graphene islands. The analysis of graphene islands over a 3 × 3 mm area of the single-crystal Cu(168) shows *T*_G_ > 0.95. For comparison we carried out the same process and analysis for epitaxial Cu(111), Cu(123), and Cu(120) (see Figure S14 in the Supporting Information), which shows *T*_G_ < 0.1 for Cu(111) and Cu(123) and *T*_G_ < 0.5 for Cu(120). This is consistent with Figure 2, i.e., our results from the polycrystalline Cu tile screening, and highlights the achieved yield increase in transfer. We note that even after long (4 weeks) oxidation, epitaxial Cu(111) was not fully oxidized, consistent with previous research.^21^ This implies that, for graphene grown on Cu(111), harsher and more damaging oxidation treatments are required to oxidize to the same standard as on Cu(168). This underscores our argument of the superior yield/quality balance achievable for such higher-index Cu orientations.

In order to highlight achievable graphene quality, we use Hall-bar devices based on widely used h-BN heterostructure encapsulation, to avoid well-known substrate, particularly SiO_2_, dependent scattering effects^42^ and to allow a direct measurement of mobility. For this, we follow previous literature,^8,9^ using a stamp terminated with a h-BN flake to cleanly transfer the graphene from oxidized CSS Cu(168) (see Experimental Details and Figures S8 and S12 in the Supporting Information for fabrication details). Figure 5d shows both the graphene island on the Cu(168)/CuO_2_ and the h-BN flake on Si/SiO_2_ used for subsequent device fabrication. Figure 5b shows a Raman 2D peak width map representative of an unencapsulated graphene island transferred on Si/SiO_2_. The Raman 2D peak width Γ_2D_ is empirically and theoretically linked to both the quality of the graphene and the effects of its support and interfacing,^43,44^ including nanometer-scale strain variations.^34^ Combined with the OM data of Figure 5a, the map highlights consistent quality across the graphene area. Figure 5e shows a map of Γ_2D_ of graphene-encapsulated in h-BN. Some folds and bubbles are seen, typical of this style of fabrication. Outlined in black and red in Figure 5e are two Hall-bar-device footprints in the most homogeneous regions, which show a mean Γ_2D_ value of 16 cm^–1^. Figure 5c compares the Raman Γ_2D_ values between graphene on SiO_2_ and on encapsulation in h-BN. This Γ_2D_ histogram shows that the h-BN-encapsulated graphene has significantly lower 2D peak widths than the graphene on Si/SiO_2_ (with mean Γ_2D_ values of 32 and 16 cm^–1^, respectively) and reflects typical values of exfoliated graphene found in the literature^43^ and other state-of-the-art pickup techniques.^7,8^ The ability to mechanically directly delaminate graphene with a h-BN stamp further highlights the effective decoupling of the graphene via interfacial Cu oxidation: that is, the Cu_2_O decreases the adhesion of the graphene to the growth substrate to below that of a graphene/h-BN interface, not requiring an intermediate wet transfer such as in other state-of-the-art encapsulation techniques.^7^Figure 5f shows the measured charge carrier mobility, μ, as a function of carrier concentration at room temperature. The devices show consistent performance with a mobility of 42.1 × 10^3^ cm^2^/(V s) at 1 × 10^12^ cm^–2^, indicating a quality on par with those of state-of-the-art exfoliated graphene and previously reported best results for CVD graphene.^7,8^

## Concluding Remarks

High-quality graphene grown on substrates that are incompatible with further processing techniques does not answer the question of how to bring the promised performance of graphene to industry and high-value-added applications. Here we have applied a holistic, data-driven approach to process optimization, utilizing fast-screening descriptors across the entirety of process steps for growth and transfer for the graphene–Cu model system. Our IPF overlay methodology allowed us to identify clear advantages of hitherto unexplored higher index Cu orientations. The increase in yield for the dry transfer of isolated CVD graphene islands shown here is essential for the many ongoing efforts to automate^45^ and accelerate a device assembly that relies on heterostructures of increasing complexity, including stacking angle or designer (meta) materials. Our approach is readily adaptable to many other catalyst–2D material systems, e.g. WS_2_/Au,^46^ h-BN/Cu,^47^ and h-BN/Pt,^48^ that are held back by analogous challenges. We anticipate the introduced high-throughput IPF-based methodology to become a potent platform to explore the many hitherto not well understood orientation dependences of chemical reactions and physical effects confined between a 2D layer and metal/substrate,^49,50^ as well as emerging epitaxial systems^46−48,51^ across many related material systems, with additional relevant descriptors easily being added.

## Experimental Details

### Plotting

The IPFs shown in this work contain regions, colored according to a provided color map, which correspond to the associated data point’s Voronoi cell, where all points in that cell are closer to the contained data point than any other. This is done in the absence of a continuum of data to clearly present regions of interest and represent the computational methods described elsewhere. It is noted that large cells are not necessarily representative of the areas they cover, and for clarity the position of each data point is clearly indicated in each cell in all IPFs in this work.

### Graphene Growth

Graphene growth was done using previously defined CVD parameters,^52^ consisting of oxidizing the Cu surface at 200 °C for 30 min, heating in a BM Pro 4′′ CVD reactor (base pressure 4 × 10^–2^ mbar) to approximately 1065 °C, where it is kept for all processes, annealing in Ar (650 sccm; 50 mbar) for 30 min, and annealing in H_2_ and Ar (100:500 sccm; 50 mbar) for 60 min followed by Ar, H_2_ and CH_4_ (0.32:64:576 sccm; 50 mbar) for 5 min to grow graphene islands. The reactor was cooled down at the base pressure with no gas flow.

### Graphene Orientation Mapping

Graphene orientation mapping was carried out on continuous graphene (aforementioned gas ratios, growth time extended to 1 h), where the sample was then exposed to H_2_ and Ar (170:470 sccm; 50 mbar) immediately after growth for 20 min. This yielded small (∼5–10 μm diameter) holes with a hexagonal shape. SEM was then used to spatially map all holes over the Cu tile: approximately 7000 SEM images at 1024 × 786 resolution at 600× magnification. These images were then binarized, stitched, and processed as detailed in Figure S4 in the Supporting Information to measure the angle of the etched hole, which was then linked spatially to the EBSD map to bin these measurements into Cu orientations. The orientations of graphene in each Cu orientation were then processed into a frequency density plot and the number or orientation was dictated by the number of peaks found by the SciPy Python package’s “find_peaks” function.

### Mechanical Delamination

Mechanical delamination was done using PVA for the systematic “tile” studies: 7 g of PVA (8000–10000 MW, 80% hydrolyzed; Sigma-Aldrich) and 3 g of PVA (85000–124000 MW, 87–89% hydrolyzed; Sigma-Aldrich) were mixed with 40 mL of DI water and stirred at 80 °C until fully dissolved. Approximately 0.1 mL cm^–2^ was placed on a removable support and dried at room temperature in a clean-room environment. Figure S8 in the Supporting Information outlines the peeling process. The PVA film was then placed onto the dried graphene/Cu at 120 °C to soften the PVA film, allowing it to adhere to the graphene and conform to the surface. The PVA/graphene was removed from the Cu at room temperature and placed onto Si/SiO_2_ at 120 °C and left for 1 min. Once the PVA was cool, the PVA/graphene/substrate was placed in DI water at 80 °C for >24 h to dissolve the PVA. The fraction of graphene transferred for each crystallographic orientation, *T*_G_, is calculated by summing the areas of graphene after transfer and taking this as a ratio with those areas that contained graphene prior to transfer:
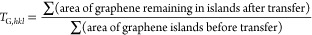
Areas were calculated by counting the number of pixels that contained graphene and scaling by the spatial dimension of each pixel.

### Dry Transfer with h-BN

Dry Transfer with h-BN was done as shown in previous literature^9^ using a stamp consisting of 13% PVA and 50 K PMMA. The polymers were spin-coated onto a glass slide at 1000 rpm and heated at 110 °C for 10 min. Subsequently, h-BN was exfoliated first using Minitron 1008R tape multiple times to decrease the thickness of crystalline h-BN and then brought into contact with the stamp. A h-BN flake of an appropriate thickness was located using confocal microscopy, and a small area was cut. This stamp was then placed onto a Gel-pack polysiloxane based support layer, such as PDMS, and glass slide. The glass/PDMS/PVA/PMMA/h-BN could then be brought into contact with graphene on the preoxidized copper substrate. After picking up the graphene, it was placed onto h-BN already exfoliated onto SiO_2_.

### Raman Spectroscopy

Raman spectroscopy was done on a Si/SiO_2_ substrate using a Renishaw InVia system at 20× magnification using a 532 nm laser (at 10% laser power) with counts accumulated over 1 s. The Si/SiO_2_ substrate was leveled prior to measurement to ensure a consistent focus prior to the batch measurements across the 1 cm^2^ sample. Each of the ∼400 spectra in each of 127 maps were fitted using separate Lorentzian profiles for the D, G, and 2D peaks. Spectra corresponding to areas where there is little/no graphene were discarded before statistics were formulated to minimize noise, defined as spectra below a threshold number of counts (400 counts in this work). Data sets with less than 10 accepted spectra were discarded to ensure a reasonable sample size per map and prohibit noise from significantly influencing the results. The values shown in the main text correspond to the statistical mean values after fitting and filtering of data. For the Raman spectroscopy studies on graphene on Cu we used a Witec alpha300R Raman imaging microscope, and the spectra were obtained with a 50× objective, equipped with a *x*–*y*–*z* DC piezo stage with the positions manually correlated to the crystallographic orientation. For excitation a 457 nm laser was used to limit the influence of the Cu background.^53^ The Raman maps were sampled before and after oxidation, as shown in Figure S10 in the Supporting Information. The maps were further processed to average values that could be correlated with the Cu crystallographic orientation, as shown in Figure S11 in the Supporting Information.

### Optical Microscopy

Optical microscopy was used to stitch together images using a 10× objective to create a final 16353 × 15752 pixel image. To remove the nonuniform contrast and brightness in the final image, a global background removal was used to remove the horizontal and vertical contrast profiles present in the stitched image that this nonuniformity caused
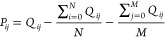
where *P*_*ij*_ is the value of the new pixel in each color channel, *Q*_*ij*_ is the original pixel, and *N* and *M* are the height and width of the image in pixels, respectively. This homogenized the image and provided a means to globally compare the contrast due to oxide of individual islands.

### Electron Backscatter Diffraction

Electron backscatter diffraction (EBSD) maps were created with a FEI Nova NanoSEM instrument at 30 kV with a 500 μm aperture. The sample was tilted to 70° approximately 17 mm from the pole piece, with the EBSD detector screen being approximately 20–25 mm from the sample. The EBSD was calibrated and optimized for Cu patterns to ensure a successful fit rate of close to 100%. The individual grains from these maps were then identified, and the (directional) mean Euler angles were used to create the larger stitched map used in this study. In this work EBSD was conducted on the Cu tile after the growth, oxidation, and peeling of the graphene.

### Interfacial Oxidation

Interfacial oxidation was achieved using water vapor as shown in Figure S2 in the Supporting Information. This consisted of heating water in the base of a closed desiccator on a hot plate to 70 °C, translating to a sample temperature of approximately 30 °C on the sample stage with a measured humidity of ∼99%. An angled glass plate was placed over the Cu substrates to prevent any water condensate from contaminating the samples. The oxidation time used in this work was 4 days, apart from the direct on Cu RS data (Figure 2d and Figure S3a in the Supporting Information), where the time period was 2 days. This was chosen, as it was observed that after this time the interfacial oxidation was no longer progressing at a noticeable rate on difficult to oxidize facets, and it can be seen that the oxide IPF shown in Figure 2 matches that of previous work^21^ over much longer time frames. The relative extent of interfacial oxidation, in this work referred to as *O*_G_, is calculated based on the optical contrast (the variation in recorded intensity by the microscope’s CMOS camera, with RGB values ranging from (0,0,0) to (255,255,255)) extracted from OM images. This takes all pixels within all islands of graphene on a particular Cu orientation and takes the mean of these values to give the initial *O*_G_ value. As *O*_G_ is only relatively measured by OM contrast, the whole data set is scaled between 0 < *O*_G_ < 1. Ellipsometry measurements, indicative of the average oxide thickness, strongly correlate to *O*_G_ and are shown in Figure S5 in the Supporting Information. The inhomogeneity of the oxide on each Cu orientation is defined as the coefficient of variation (*C*_v_) of the relative oxidation postnormalization with respect to the whole data set
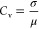
where σ, the standard deviation, and μ, the mean, are both taken from the subset of data corresponding to a given crystallographic orientation of Cu. This measure was used to compensate for the significant differences in mean oxidation levels between Cu orientations and highlight inhomogeneity within those levels to enable comparison to other orientations.

### Scanning Electron Microscopy (SEM)

Scanning electron microscopy (SEM), with a Zeiss Gemini SEM instrument, was used to map the SiO_2_ substrate after transfer and the Cu tile after growth of graphene and etching. The samples were first leveled such that stage movements did not result in any change of focus of the substrates, and then the manufacturer-provided API was used to automate stage movement and take approximately 7000 images at a magnification of 600× over 1 × 1 cm^2^. These images were then stitched and binarized to reveal areas where graphene was present, which could be used for the extraction of *T*_G_.

### Close-Spaced Sublimation (CSS)

Close-spaced sublimation (CSS) was done in a BM Pro 4′′ CVD reactor (base pressure 4 × 10^–2^ mbar) following previous work.^15^ Single-crystal MgO(168), single side polished (SurfaceNet GmbH), 1 × 1 cm^2^ crystals were rinsed in acetone and then IPA (1 min each) before being dried in N_2_ and loaded into the BM Pro reactor. The MgO was placed 1 mm away from a planar polycrystalline Cu source (Alfa Aesar; 1 mm thick; 99.9%). The source was then heated to 1075 °C for 60 min, while the MgO substrate was approximately 950 °C. This resulted in the epitaxial sublimation of Cu onto the MgO with the desired Cu orientation.

### Hall-Bar Devices

Hall-bar devices were fabricated with dry-transferred Gr, originating from Cu(168), and fully encapsulated by h-BN as in previous work.^9^ The Hall-bar structures were defined in homogeneous regions with the lowest Γ_2D_ with values of around 16 cm^–1^, indicating very small nanometer-scale strain variations of the graphene layer.^34^ Further processing used electron beam lithography to define the shape, aluminum deposition to protect the region of interest, and reactive ion etching with SF_6_ to etch away the undesired material. A subsequent lithography step was performed to define contacts, and edge contacts were finally contacted with Cr/Au (5 nm/75 nm). For electrostatic gating, highly p-doped Si was used, covered by a layer of 300 nm thick silicon oxide. The device geometry had a channel length of 4 μm and a channel width of 3 μm. The h-BN/Gr/h-BN Hall-bar device sat on top of the silicon oxide layer, and the h-BN had a thickness of roughly 20 nm.

### Electrical Transport Measurements

Electrical transport measurements were performed with the Hall-bar devices in a vacuum-pumped system. Standard lock-in techniques were used to measure the four-terminal resistance as well as Hall voltage and Hall conductivity. The charge carrier mobility μ as a function of charge carrier concentration *n* was calculated using the Drude formula σ = *ne*μ, where σ is the electrical conductivity. The electron mobility was extracted at a temperature of 300 K and a carrier concentration of *n* = 1 × 10^12^ cm^–2^ to give 42.1 × 10^3^ cm^2^/(V s).

## Supporting Information Available

Additional analysis and evidence on the methodology and additional experimental results, including oxidation, dry transfer process, EBSD mapping, XPS, AFM, and Raman analysis (PDF)

## Abbreviations

CVD: chemical vapor deposition
2DM: 2D material
h-BN: hexagonal boron nitride
IPF: inverse pole figure
PVA: poly(vinyl alcohol)
OM: optical microscope
SEM: scanning electron microscope
EBSD: electron backscatter diffraction
RCE: rotating compensator ellipsometry
IPA: isopropyl alcohol
XPS: X-ray photoemission spectroscopy
IE: imaging ellipsometry
