# Supplementary material for: Putting High-Index Cu on the Map for High-Yield, Dry-Transferred CVD Graphene
Source: ACS Nano. 2023 Jan 3;17(2):1229–38. doi: 10.1021/acsnano.2c09253 (PMC9878973; doi:10.1021/acsnano.2c09253)
Supplement: Supplementary file 1 — nn2c09253_si_001.pdf [file nn2c09253_si_001.pdf]

# Supplementary Information: Putting high-index Cu on the map for high-yield, dry-transferred CVD graphene

*Oliver J. Burton[1‡*], Zachary Winter[2‡*], Kenji Watanabe[3], Takashi Taniguchi[4], Bernd Beschoten[2], Christoph Stampfer[2,5], Stephan Hofmann[1]*

[1]Department of Engineering, University of Cambridge, Cambridge CB3 0FA, United Kingdom

[2]2nd Institute of Physics A and JARA-FIT, RWTH Aachen University, 52074 Aachen, Germany

[3]Research Center for Functional Materials, National Institute for Materials Science, 1-1 Namiki Tsukuba, Ibaraki 305-0044, Japan

[4]International Center for Materials Nanoarchitectonics, National Institute for Materials Science, 1-1 Namiki Tsukuba, Ibaraki 305-0044, Japan

[5]Peter Grünberg Institute (PGI-9), Forschungszentrum Jülich, 52425 Jülich, Germany

[1] EBSD grain detection and stitching

[2] Humidity-based oxidation chamber schematic

[3] Relative oxidation level (OG) of Cu under graphene

[4] Orientation mapping of Gr on Cu

[5] Ellipsometry measurement of average oxide thickness

[6] Raman spectroscopy of Gr after transfer, 2D and G peak widths

[7] XPS of Gr/Cu, post interfacial oxide formation

[8] Schematic of tile processing for the high throughput study

[9] AFM measurements of Cu and $Cu_2O$ regions

[10] Raman sampling on copper before and after oxidation

[11] Method for extracting Raman data averages

[12] Dry-transfer process

[13] Comparison of oxidation under graphene and on uncovered Cu

[14] Single crystal Cu graphene oxidation and transfer

## S1: EBSD grain detection and stitching

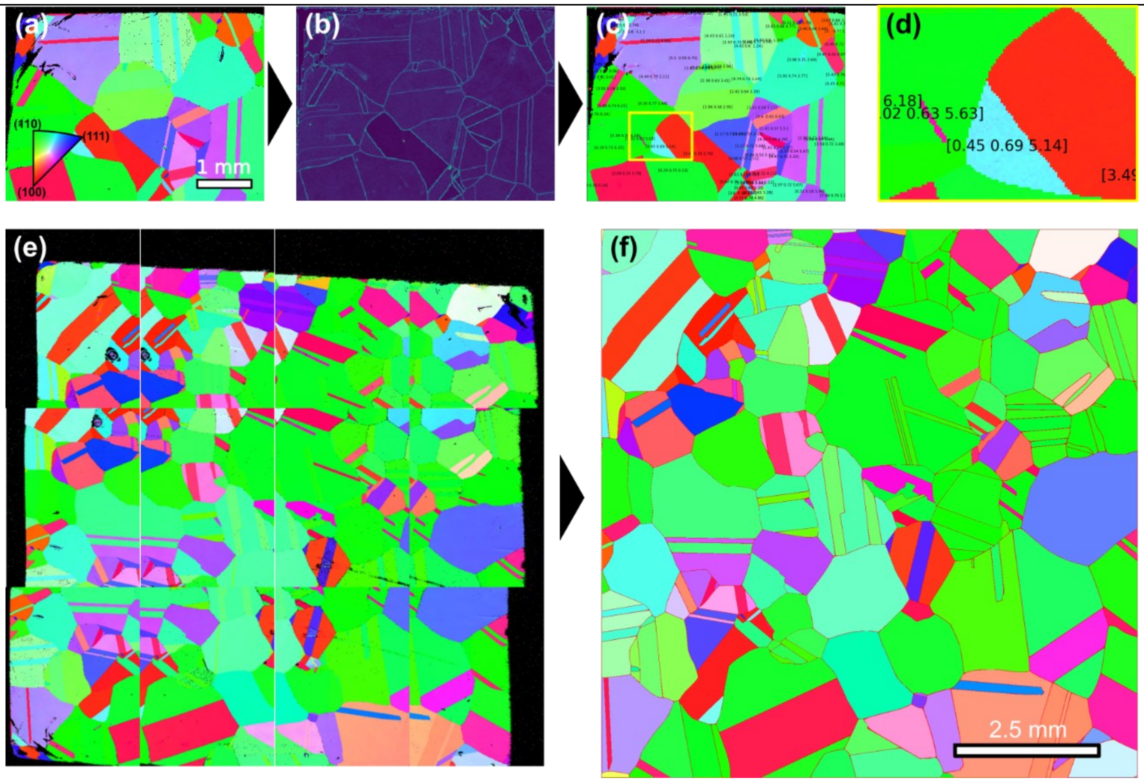


Figure S1: (a) the Z-IPF map of representing the raw data accumulated from the EBSD mapping. (b) The data represented in (a) after a mean difference operation has been used to show the presence of grain-boundaries and identify individual crystallographic orientations. (c) Directional mean Euler angles of the individual Cu grains as identified in (b). (d) The yellow region shown in (c). (e) The unprocessed map consisting of 12 ill-aligned sub-maps. (f) The finished spatial orientation map (represented here as z-IPF colormap) used for this study.

Figure S1 shows the general process for EBSD map stitching into a larger map corresponding to an area spanning the 1x1 cm Cu tile used in this work. A buffer zone of width > 500 μm corresponding to larger than the largest detected island (graphene islands were of mean diameter

and standard deviation: 92 ± 24 μm) was used to remove any potentially erroneous data points corresponding to e.g. graphene nucleating on one orientation and growing onto another.

## S2: Humidity-based oxidation chamber schematic

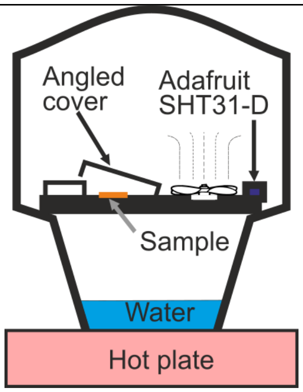


Figure S2: Schematic representation of the humidity-based oxidation apparatus and approximate sensor positioning. The oxidation was achieved by heating water in the base of closed desiccator on a hotplate to 70 °C, translating to a sample temperature of approximately 30 °C on the sample stage. A fan was placed on the sample stage to distribute water vapor and bring a stable internal humidity to > 99 %. Temperature and humidity were measured using an Adafruit SHT31-D connected to a Raspberry Pi. The entire set-up was placed in a temperature stabilized room to decrease internal temperature fluctuations. The sample was then covered with an angled glass plate to avoid any potential condensation falling onto the copper/graphene substrate.

## S3: Relative oxidation level ($O_G$) of Cu under graphene

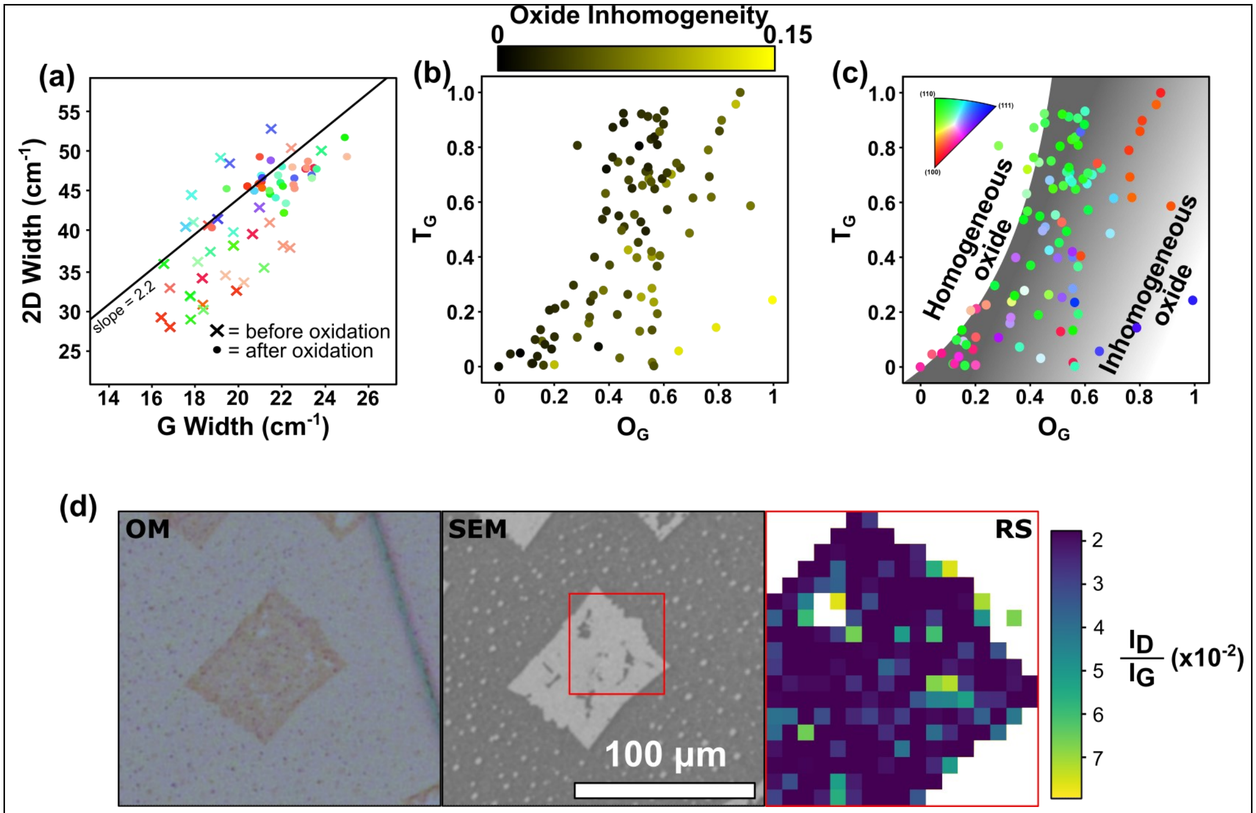


Figure S3: (a) The average Raman 2D peak width against the G peak width for graphene on Cu before (x) and after (o) oxidation. The color of this scatter plot is linked to the crystal orientation of the Cu that the graphene measurement was taken, according to the z-IPF inset in (c). (b) A reproduction of the data shown in Figure 2(d) to allow for comparison: a scatter plot of $T_G$ vs $O_G$ for all orientations measured, with the color of each point indicating the oxide inhomogeneity as defined in the methods. (c) The same data as in (b), where each point has been colored to indicate the crystallographic orientation. The shaded region is a guide to the eye to help indicate some additional trends relating to oxide homogeneity and transferability. (d) Example images of the island before (OM) and after (SEM) transfer, with a Raman map after transfer shown.

Figure S3 reinforces the data displayed in Figure 2 in the main text: with S3(c) showing some of the individual trends in oxidation with rough sections of the crystallographic space: towards Cu(111) (indicated by blue) we note that these have a higher mean oxide thickness yet much lower transferred proportions, though the general trend of increasing oxide leading to increased transfer remains the same as in other orientations. Figure 3(d) highlights that the different $I_D/I_G$ of different Cu orientations is largely due to contributions from the cracks or areas where small amounts of graphene have not transferred and damaged the surrounding region.

## S4: Orientation mapping of Gr on Cu

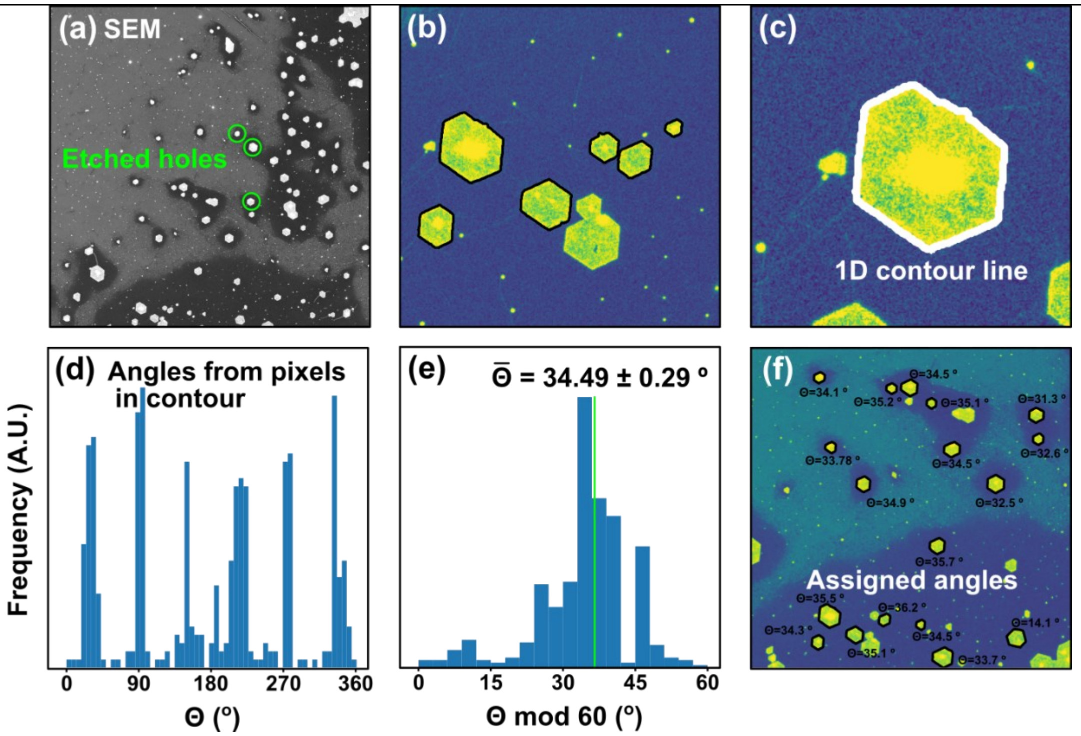


Figure S4-1: Island identification and principle angle determination. (a) SEM image of the etched holes in continuous graphene. (b) contrast-based contours with circularity > 0.7. (c) Example close-up image of etched hole contour line. (d) A histogram of the angles of points on the contour relative to neighboring (6 pixels along) points. (e) The angles from (d) condensed by a modulus function considering the constant angular offset of one side of a hexagon to another, allowing for the mean angle and standard error to be calculated using directional statistics. (f) A plot of the etched holes, showing which ones have been identified and the principal angles assigned to them.

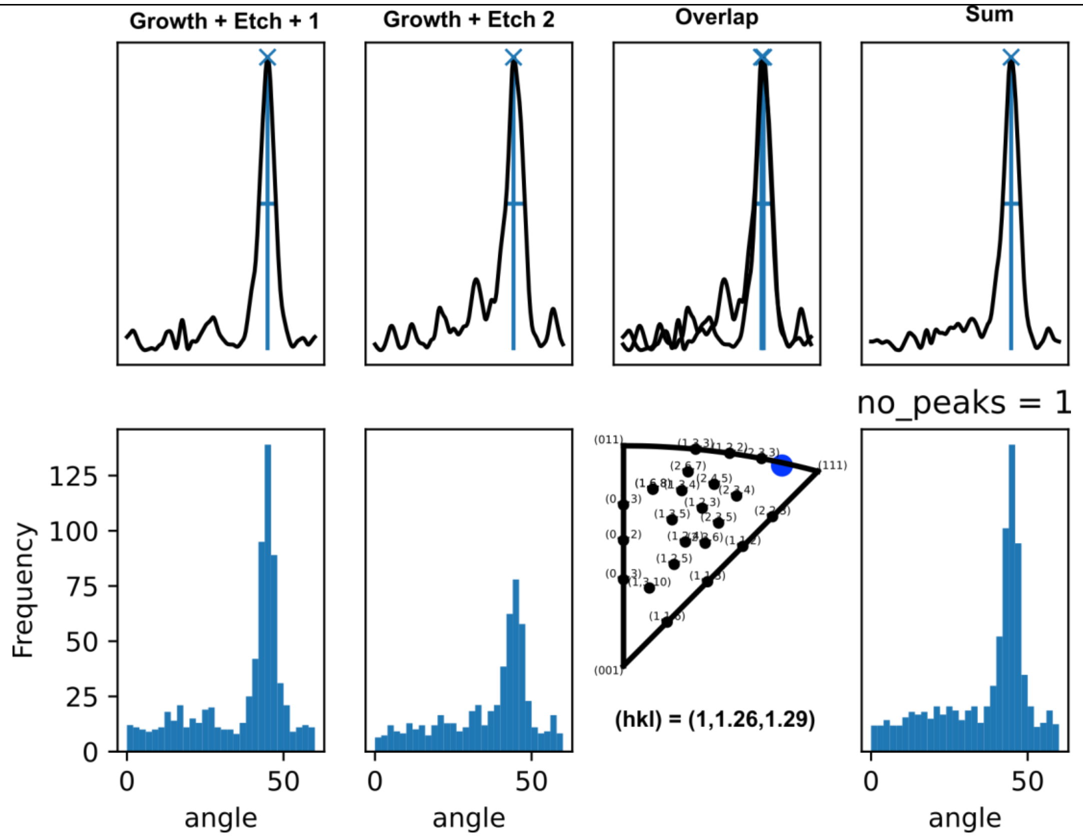


Figure S4-2: Example of the formation of a single segment of the plot on Figure 4: Two experimental datasets were created by the repeated growth of continuous film and subsequent etching detailed in previous literature[1], done on the same tile to both demonstrate experimental repeatability between growth and etches, and to increase signal strength. The resulting SEM image maps over 1x1 cm were processed using homebuilt code to extract the orientation of etched hexagonal domains. This data was then mapped on to the EBSD data to give each etched hexagonal domain detected an orientation and crystallographic orientation of the Cu. Etched holes were then grouped into datasets of orientation (i.e. each grain on the Cu), constructed into a histogram and associated density plot, where any peak(s) could be identified. As shown in the example case, the two independent datasets overwhelmingly correspond to each other, with each having one peak and the same position, and demonstrates

the repeatability of the orientation measurement technique. The final number of peaks identified is taken from the combination of both datasets and plotted in Figure 4.

To map the number of orientations of graphene on each Cu facet, a graphene etching process[1,2] was used to reveal the zig-zag edges of the graphene on the Cu, in the form of hexagonally etched holes, shown in Figure S4-1(a). Figure S4-1,2 shows the process of edge detection, inter-pixel measurement of that edge, binning and moduli to produce a histogram of measured angles (computationally). This was then analyzed with directional statistics to give a mean angle and associated standard deviation which was used to filter those etched holes or other sources of edges that were not hexagonal. Circularity and area were also used to minimize the effect of erroneous contributors to the statistics. **Circularity is defined here as:** $\boldsymbol{Circularity = \frac{4\pi * area}{perimeter^2}}$ **as a measure of how circular a specific shape is. The circularity varies between 0 and 1, with a regular or perfect hexagon measuring at 0.907.** The end result, after processing as detailed in the Methods section, was a histogram of etch hole angles that could be fitted with peaks to find the number of orientations present on a particular Cu orientation.

**S5: Ellipsometry measurement of average oxide thickness**

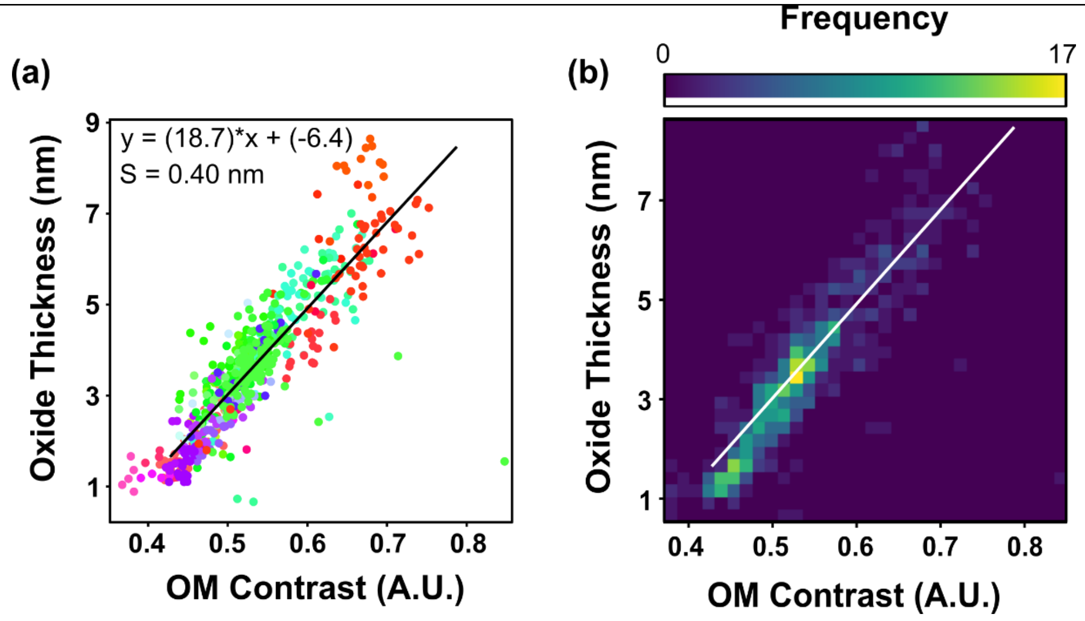


Figure S5: Correlation between OM contrast measurements and RCE determined average oxide thickness. (a) z-IPF colored scatter plot showing the relative OM contrast measurements plotted against RCE measurements of OM thickness. (b) the same as (a), where the plot is a density based heatmap where the color bar maps to the frequency of points in that bin. A least-squares linear fit has a correlation coefficient of 0.88, a strong correlation, and a standard error of 0.4 nm, illustrating that OM contrast is a reasonable method for relative oxide thickness measurement in the Cu system.

To demonstrate the correlation of OM image contrast and average oxide thickness measurements through rotating compensator ellipsometry (RCE), we take several local psi and delta maps at wavelengths of 400, 575 and 750 nm. We fit these maps with the supplied Sopra public database model (for n and k) for $Cu_2O$ and extract a spatial map of $Cu_2O$ thickness. The Psi, Delta and

thickness maps each have a field of view of 500x581 μm, corresponding to a resolution of 349x406 pixels. We then find the graphene domains in each thickness map and compare the average value within each graphene domain (i.e., each data point is the average of the values within a graphene domain).. The Cu average oxide thickness was fitted with Accurion's $Cu_2O$ model. This was done to compensate for a much lower spatial resolution given by the ellipsometry measurements. The results are summarized in Figure S5, showing a clear correlation and linear fit between the ellipsometry derived average oxide thicknesses and the contrast derived relative thicknesses from OM. It is noted that when the Cu tile was mapped using the ellipsometers in-built software, there was not a linear spatial relationship between the ellipsometry data and the OM, SEM or RS measurements, thus this could not be used for the large-scale correlation as the other datatypes. It did however provide a range for the maximum and minimum levels of interfacial oxidation, between 0 and 10.2 nm, which given the linear and monotonic relationship shown here could be used in place of the relative oxidation measure ($O_G$) in Figures 2 and S3.

**S6: Raman Spectroscopy of Gr after transfer, 2D and G peak widths.**

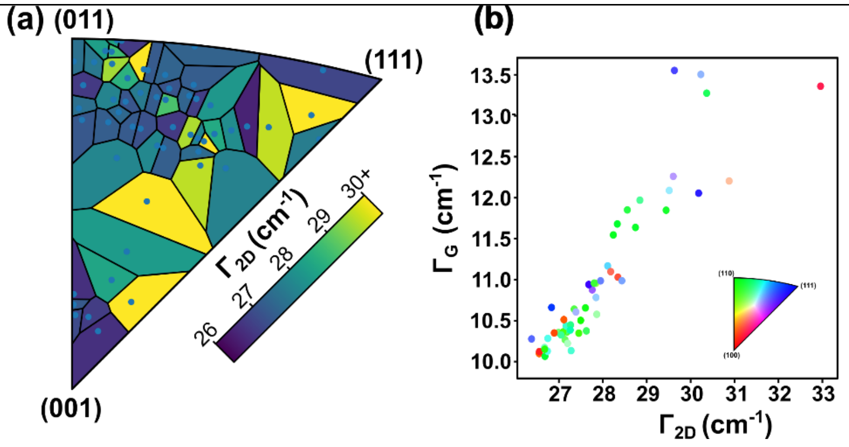


Figure S6: (a) The RS 2D peak width ($\Gamma_{2D}$) of graphene transferred onto $SiO_2$ with PVA as a function of crystallographic orientation of Cu. (b) A scatter plot of the $\Gamma_{2D}$ vs $\Gamma_G$, using the same data points as (a), where the color of each plotted point corresponds to the crystallographic orientation of the Cu the graphene was peeled from per inset IPF color reference.

Figure S6 shows the RS data plotted in an IPF map showing $\Gamma_{2D}$ as a function of the Cu orientation that the graphene was from and $\Gamma_{2D}$ vs $\Gamma_G$. $\Gamma_{2D}$ is an indicator of nanometer-scale strain variations and has been shown to correlate with the mobility of graphene-based Hall devices[3], and as such could be used as a strong indicator for the relative 'quality' of the transferred graphene along with the $I_D/I_G$ ratio shown in Figure 2.

## S7: XPS of Gr/Cu, post interfacial oxide formation

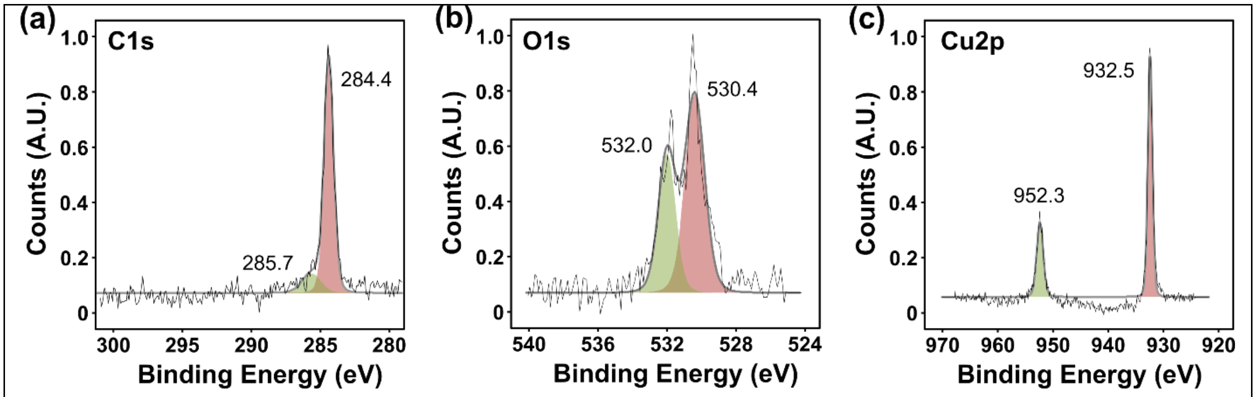


Figure S7: XPS measurements of graphene on Cu(168) after oxide formation with peak positions, determined by fitting the peaks with asymmetric Voight-like line profiles, shown adjacent to the highlighted peak. The black line indicates the experimental counts after background subtraction whilst the grey line indicates the sum of the individual fits. (a) The C1s peak with (red) carbon-carbon bonding and (green) carbon-oxygen bonding. (b) The O1s peak with (red) metal oxide and (green) metal carbonate sub-peaks. (c) The Cu2p peaks with (red) Cu2p3/2 and (green) Cu2p1/2 sub-peaks.

XPS measurements were conducted on Cu(111), Cu(120), Cu(121), Cu(122), Cu(123), Cu(236) and Cu(168) single crystals after graphene island growth and oxidation (See methods section). All samples showed near identical spectra, with graphene on Cu(168) after oxidation shown in Figure S7 as a representative example: no $Cu^{2+}$ satellites were observed (Figure S7(c)), despite copper oxide (Figure S7(b)) being present.[4] These results imply that the Cu oxide observed in this work is $Cu_2O$. Figure S7(a) shows the presence of graphitic or $Sp^2$ hybridized carbon (red), which we mainly attribute to the as grown graphene in this work; the shoulder (green) is

attributed to carbon-oxygen bonding and is likely due to adventitious carbon contamination on the surface of the sample.[4]

**S8: Schematic of tile processing for the high throughput study**

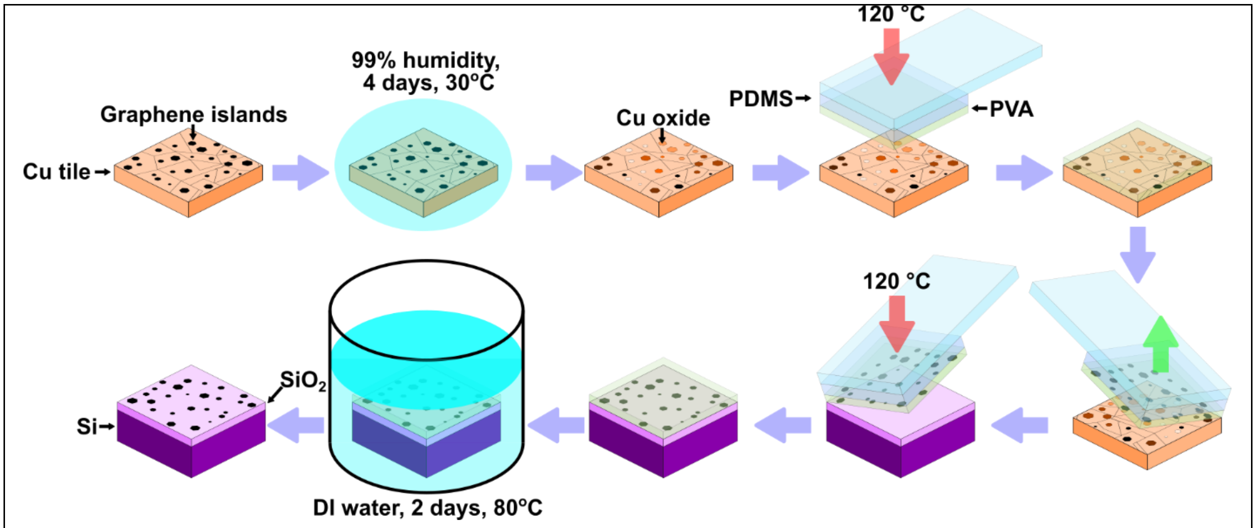


Figure S8: A Schematic of the process order for the growth of islands, interfacial oxidation, mechanical delamination and application to a target substrate.

## S9: AFM measurements of Cu and $Cu_2O$ regions

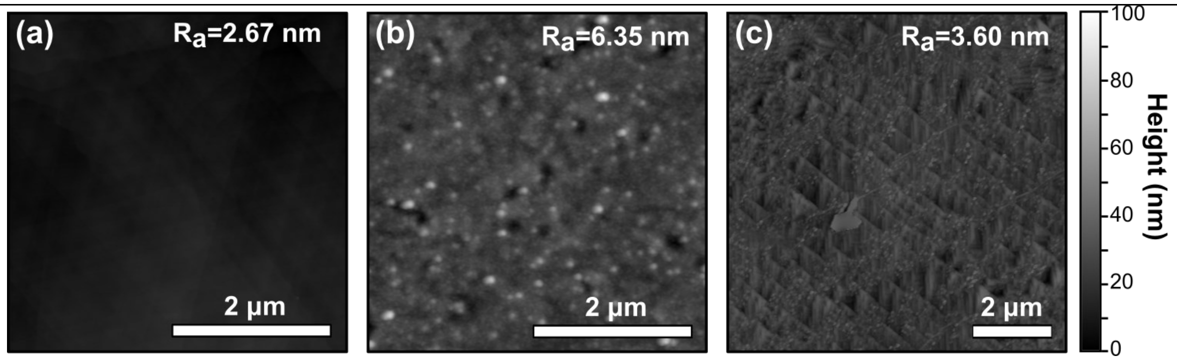


Figure S9: AFM maps before (a) and after (b) a typical oxidation process measured on graphene regions on a single crystal Cu Sample, showing the increased surface roughness beneath the graphene as a result of the oxidation process. (c) shows an AFM map of the surface of Cu(168) grown on MgO as a single crystal with no graphene after annealing, it is noted that some oxide formation is likely due to the speed at which this orientation tends to oxidize, despite less than a one hour time frame. Despite the fast oxidation, the crystal structure is apparent and shows clear step bunching. It should also be noted that a large particle in the bottom left hand corner of (c) was masked and not used in the calculation of roughness.

## S10: Raman sampling on copper before and after oxidation

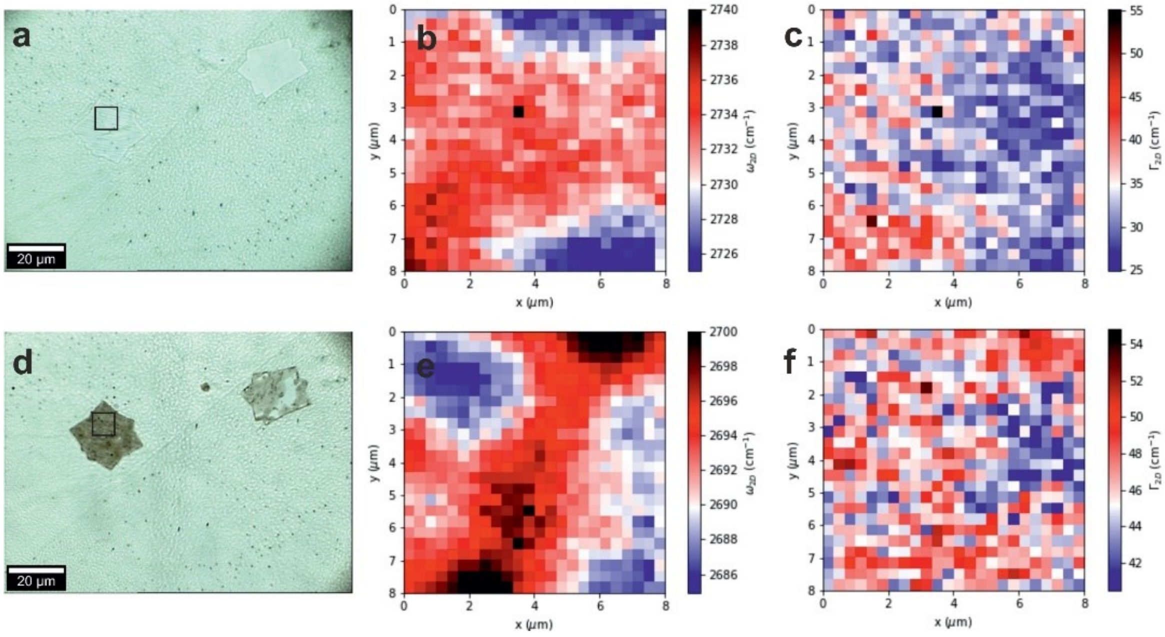


Figure S10: Example of Raman sampling for islands before (a-c) and after 4 days of oxidation on copper (d-f). Optical images of graphene islands on cooper are shown before and after oxidation in (a) and (d) and an 8x8 μm$^2$ area shows where Raman maps were taken. The 2D positions (b and d) and FWHM maps (c and d) are compared. The maps are subsequently processed as shown further in Figure S11.

## S11: Method for extracting Raman data averages

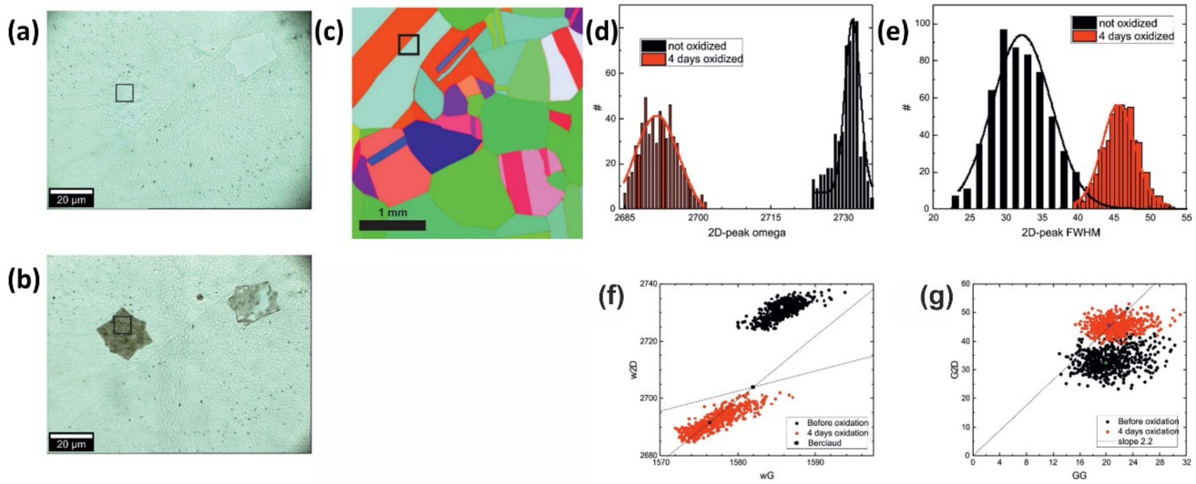


Figure S11 (a-b) show the graphene islands before and after oxidation. The copper orientation is known from EBSD measurements as shown in (c). The 8x8 μm2 Raman maps are condensed into histograms in (d-e) and the scatter plots are shown in (g-h). The average positions are plotted in Figure 2 (d) and widths in Figure S3.

## S12: Dry-transfer process

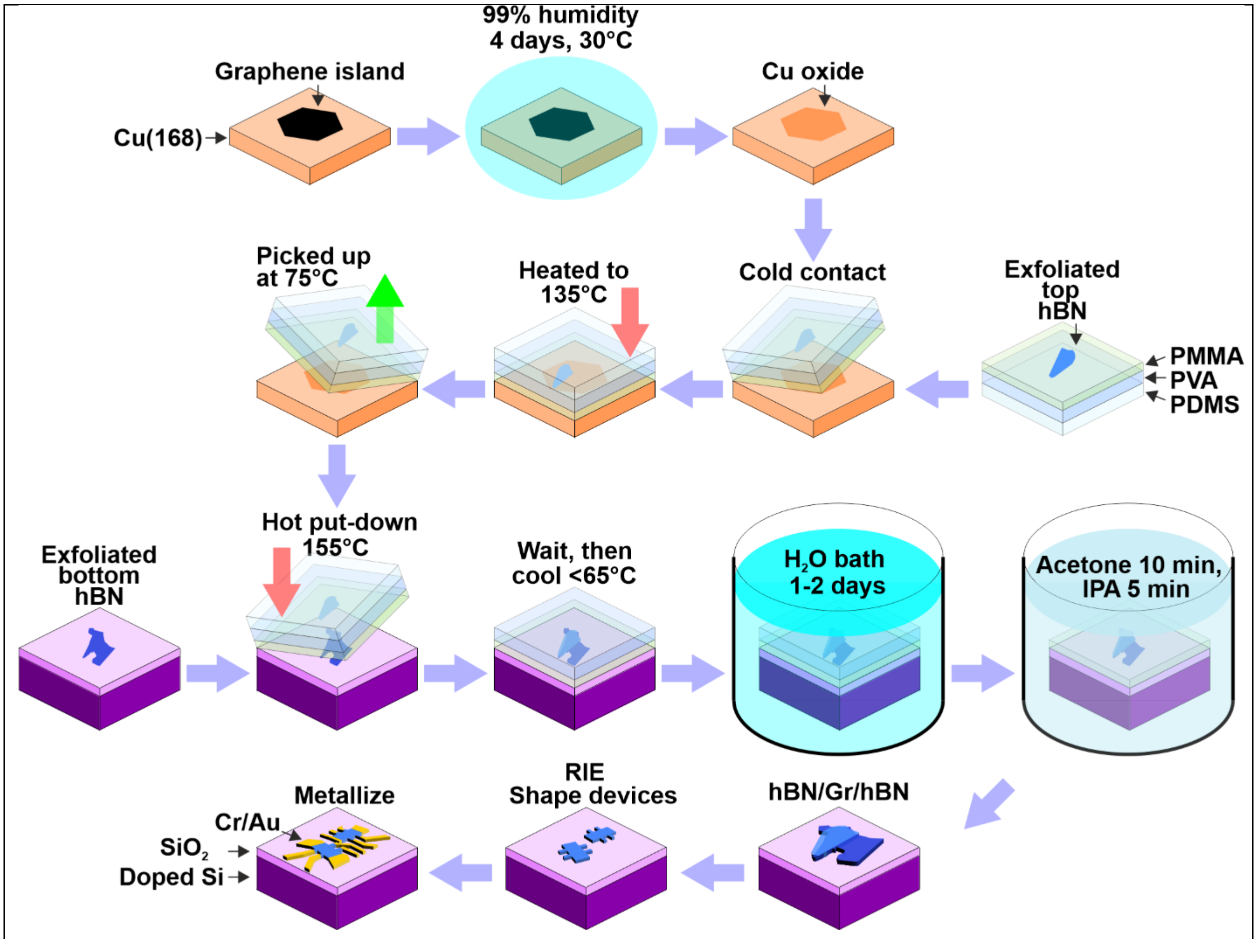


Figure S12: A schematic of the entire dry-transfer process from epitaxial Cu(168) substrate to finished Hall bar device. A single graphene island is picked-up with an exfoliated h-BN flake and placed down onto an exfoliated h-BN already on the target SiO2 substrate using the hot put-down technique. The polymers are removed and devices are structured using E-beam lithography, RIE for etching, and finally side-contacts are made using Cr/Au.

## S13: Comparison of oxidation under graphene and on uncovered Cu

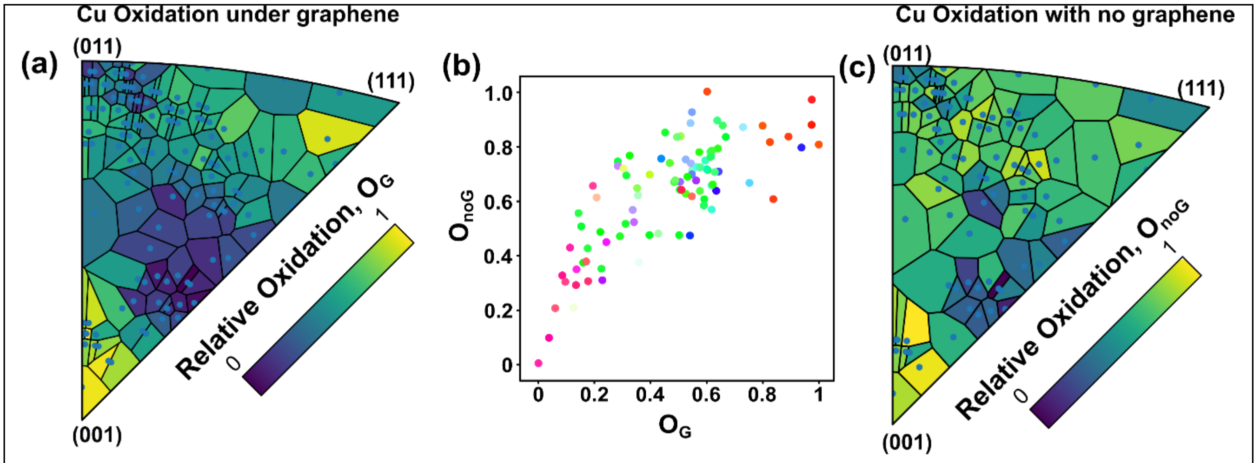


Figure S13: (a) A z-IPF showing the relative oxidation (defined in methods) of the Cu-Gr interface, $O_G$, dependent on crystallographic Cu orientation. (b) The relative oxidation of Cu facets on uncovered Cu against that of the graphene-Cu interface. This scatter plot effectively demonstrates a correlation between the oxidation of Cu uncovered and covered by graphene, with a Pearson correlation coefficient of 0.88. (c) A z-IPF showing the relative oxidation (defined in methods) of the uncovered Cu surface, $O_{noG}$, dependent on crystallographic Cu orientation.

**S14: Single crystal Cu graphene oxidation and transfer**

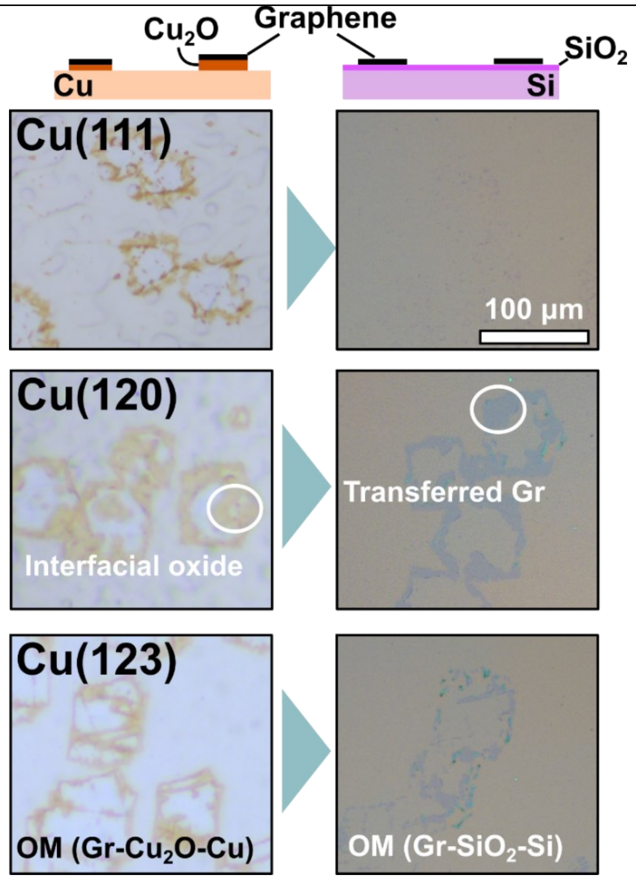


Figure S14: The oxidation of CSS deposited single crystal Cu samples with graphene grown and transferred, as per methods section. Similarities between the oxidization pattern and the transferred graphene can be clearly seen in Cu(120) and Cu(123), and again highlights the need for a consistent, homogeneous oxide. Cu(111) had only small flakes of graphene transferred, likely corresponding to the edge regions. Scale bar is the same for all images.

## References


1. Burton, O. J. *et al.* The Role and Control of Residual Bulk Oxygen in the Catalytic Growth of 2D Materials. *Journal of Physical Chemistry C* **123**, 16257–16267 (2019).
2. Burton, O. J. *et al.* Integrated wafer scale growth of single crystal metal films and high quality graphene. *ACS Nano* **14**, 13593–13601 (2020).
3. Couto, N. J. G. *et al.* Random Strain Fluctuations as Dominant Disorder Source for High-Quality On-Substrate Graphene Devices. *Phys. Rev. X* **4**, 041019 (2014).
4. Moulder, J. F., Stickle, W. F., Sobol, P. E. & Bomben, K. D. *Handbook of X-ray photoelectron spectroscopy: a reference book of standard spectra for identification and interpretation of XPS data*. *Surface and Interface Analysis* (Perkin-Elmer Corporation, 1992).
